# In vitro effects of 3 % hypertonic saline and 20 % mannitol on canine whole blood coagulation and platelet function

**DOI:** 10.1186/s12917-015-0555-x

**Published:** 2015-09-24

**Authors:** Katja-Nicole Adamik, Emmanuelle Butty, Judith Howard

**Affiliations:** Division of Emergency and Critical Care, Small Animal Clinic, Department of Clinical Veterinary Medicine, Vetsuisse Faculty, University of Bern, Laenggassstrasse 128, 3012 Bern, Switzerland; Clinical Diagnostic Laboratory, Department of Clinical Veterinary Medicine, Vetsuisse Faculty, University of Bern, Laenggassstrasse 124, 3012 Bern, Switzerland

**Keywords:** Dog, Hemostasis, Hypertonic saline, Hypocoagulation, Mannitol, Osmotherapeutics, Intracranial hypertension

## Abstract

**Background:**

Hyperosmolar therapy, using either mannitol or hypertonic saline (HTS), is considered the treatment of choice for intracranial hypertension. However, hyperosmolar agents may impair coagulation and platelet function, limiting their use in patients at risk for hemorrhage. Despite this, studies evaluating the effects of mannitol compared to other hyperosmolar agents in dogs are largely lacking. The aim of this study was to compare the in vitro effects on global hemostasis and platelet function of 20 % mannitol and 3 % HTS on canine blood.

**Methods:**

Citrated whole blood from 15 healthy dogs was diluted with 0.9 % saline, 20 % mannitol and 3 % HTS in ratios of 1:16 and 1:8. Rotational thromboelastometry (ROTEM) was used to assess clotting time (CT), clot formation time (CFT) and maximal clot firmness (MCF) following extrinsic activation (Ex-tem) and after platelet inhibition (Fib-tem). A platelet function analyzer (PFA-100) was used to assess closure time (Ct_PFA_).

**Results:**

No significant differences were observed between untreated whole blood and samples diluted with saline. Samples diluted with both mannitol and HTS were hypocoagulable compared to untreated whole blood samples. At a dilution of 1:16, no significant differences were found between any measured parameter in samples diluted with saline compared to mannitol or HTS. At a 1:8 dilution, Ct_PFA_ was prolonged in samples diluted with mannitol and HTS compared to saline, and Ct_PFA_ was prolonged more with mannitol than HTS. Ex-tem CT was increased at a 1:8 dilution with mannitol compared to HTS. Ex-tem CFT was prolonged at a 1:8 dilution with both agents compared to saline, and was prolonged more with mannitol than HTS. Ex-tem MCF was reduced at a 1:8 dilution with both agents compared to saline.

**Discussion and Conclusions:**

Data in this study indicate that both mannitol and HTS affect canine platelet function and whole blood coagulation in vitro in a dose-dependent fashion. The most pronounced effects were observed after high dilutions with mannitol, which impaired platelet aggregation, clot formation time, clot strength, and fibrin formation significantly more than HTS. Further in vivo studies are necessary before recommendations can be made.

## Background

Hyperosmolar therapy has been used for decades to treat intracranial hypertension in both human and veterinary medicine [1–3]. Mannitol has been the primary hyperosmolar agent used since the 1960s and concentrations of 15 to 20 % have been shown to have beneficial effects on intracranial pressure, cerebral perfusion pressure and cerebral blood flow, as well as on brain metabolism [3, 4]. Hypertonic saline (HTS) solutions of different strengths have been used as alternative agents to mannitol and have been shown to reduce intracranial pressure, and improve cerebral blood flow and oxygen delivery [2, 5, 6]. Indeed, numerous reports have suggested that 7.5 % HTS may be superior to mannitol for the treatment of intracranial hypertension in both humans and dogs [7–11]. However, several studies have shown that both mannitol and HTS interfere with coagulation and platelet function in humans, which may limit their use in some patients [12–15]. Proposed mechanisms of impaired hemostasis include dilutional coagulopathy, platelet dysfunction, diminished clot propagation and clot strength, as well as impaired fibrin formation [12–14]. Dogs with severe traumatic brain injury or pre-existing coagulopathies may therefore be at increased risk of hemorrhage after administration of hyperosmolar agents.

Although several studies have investigated the hemodynamic effects of hyperosmolar agents in dogs with hemorrhagic, traumatic, endotoxic, and hypovolemic shock, only few studies have investigated their effects on coagulation in dogs [16–23]. A recent in vitro study in dogs found that 7.2 % HTS and hypertonic hydroxyethyl starch impair platelet aggregation as well as parameters of global coagulation measured using rotational thromboelastometry (ROTEM) [24]. Another study found no deviation from reference intervals in platelet counts, prothrombin time and partial thromboplastin time following infusion with 7.5 % HTS combined with colloids (dextran or hydroxyethyl starch) in a canine hemorrhagic shock model [25]. However, no study has evaluated the effects of mannitol on coagulation and platelet function compared to other hyperosmolar agents in dogs, and the relative risk of impaired hemostasis following administration of these agents is largely unknown. Despite this, these agents are used in practice and dosage recommendations have been published [1, 2, 26–28]. Knowledge of the extent to which different hyperosmolar agents may impair global hemostasis or platelet function may influence clinical decisions in the selection of agents to treat individual dogs based on a perceived risk of hemorrhagic complications. The purpose of this study was to investigate and compare the in vitro effects of 20 % mannitol and 3 % HTS on canine platelet function and whole blood coagulation.

## Methods

The study was designed as an experimental non-randomized comparative in vitro study. The protocol was approved by the University of Bern and the Animal Experiment Committee of the Swiss Federal Veterinary Office (BE 110/12) and all dog owners gave written permission for blood sampling.

### Animals

Fifteen healthy, staff- and student-owned dogs were included in the study. All dogs weighed >15 kg to ensure a large vessel diameter for adequate blood flow into the blood collecting tubes. Dogs were considered to be healthy based on physical examination and unremarkable findings of a complete blood count and serum biochemical analyses. Dogs were excluded if there was a history of a bleeding disorder, chronic or current illness, or the administration of drugs within 4 weeks prior to the study. The dogs had a mean age of 3.8 years (range, 1.0 to 9.5 years). Eight dogs were female (four sexually intact; four spayed) and seven were male (five sexually intact; two castrated). Food was withheld from all dogs for 10 h prior to blood collection.

### Sample collection

Blood (13.4 ml) was obtained from each dog by lateral saphenous venipuncture using a 21-gauge needle and distributed directly under continuous blood flow into blood tubes in the following order: 1 ml into a lithium-heparin tube (Sarstedt AG, Switzerland) for biochemical analyses, 3.8 ml into each of three buffered 3.8 % sodium citrate tubes (S-Monovette for PFA-100, Sarstedt AG, Switzerland) for platelet function analysis (PFA-100) and rotational thromboelastometry (ROTEM), and 1 ml into an EDTA tube (Sarstedt AG, Switzerland) for a complete blood count. Immediately following collection, the tubes were inverted carefully to ensure adequate mixing. The blood was maintained at room temperature and all analyses were carried out within 3 h of blood collection (immediate analysis of all samples was not possible due to the number of samples and time necessary for ROTEM analyses).

### Sample preparations and measurements

Measurement of baseline values for PFA-100 and ROTEM were performed in undiluted blood samples from each dog. In addition, aliquots of each blood sample were diluted with 3 % HTS (3 % sodium chloride solution, Dr. Grogg Chemie AG, Switzerland), 20 % mannitol (20 % mannitol, Laboratorium Dr. G. Bichsel AG, Switzerland), and saline (0.9 % sodium chloride solution, Fresenius Kabi AG, Switzerland) in dilutions of 1:16 (6.25 vol. % dilution, i.e. 1 part study solution and 16 parts blood) and 1:8 (12.5 vol. % dilution). All samples were mixed gently and incubated at room temperature prior to analyses. All PFA and ROTEM analyses were performed as single measurements.

### Whole blood coagulation

Whole blood coagulation was analyzed by rotational thromboelastometry (ROTEM®, TEM Innovation GmbH, Munich, Germany) according to the manufacturer’s instructions using methods previously described for canine samples [29]. Briefly, a 300 μL sample of citrated whole blood was added to the clotting reagents in the pre-warmed cuvette using the supplied electronic pipette. The assays used were Star-tem, Ex-tem and Fib-tem (Tem Innovation GmbH, Munich, Germany): After recalcification with Star-tem reagent, coagulation was initiated by the extrinsic pathway by addition of Ex-tem reagent (recombinant tissue factor), and by the extrinsic pathway by addition of Ex-tem reagent with re-calcification and platelet inhibition by cytochalasin D using the Fib-tem reagent. The clotting reactions were assessed using the manufacturer’s data processing software. Data obtained included clotting time, CT (the time from initiation of the reaction until onset of clotting), clot formation time, CFT (the time from the onset of clotting until clot firmness reaches amplitude of 20 mm) and maximal clot firmness, MCF (the maximum amplitude of the thromboelastogram curve).

### Platelet function

Platelet function was analyzed using a platelet function analyzer (PFA-100®, Siemens Healthcare Diagnostics AG, Zurich, Switzerland). The analysis was performed using methods previously described for canine blood [30, 31]. Briefly, collagen/adenosine-5-diphosphate (CAPD) cartridges (Dade® PFA Collagen/ADP Test Cartridge, Siemens Healthcare Diagnostics, AG, Zurich, Switzerland) were warmed to room temperature and 0.8 mL citrated whole blood was added. The sample was then aspirated under constant vacuum from a reservoir through a capillary and a microscopic aperture in a membrane, which is coated with collagen and ADP. The PFA-100 measures the closure time (Ct_PFA_) in seconds, the time needed for occlusion of the aperture by platelet plug formation.

### Statistical analyses

All analyses were performed using commercial statistical software (MedCalc version 12.7.7, MedCalc bvba, Ostend, Belgium). Data sets were evaluated for normality using the D’Agostino-Pearson test and by examining normal plots. As some data sets were not normally distributed and could not be transformed to normality, differences between data in undiluted samples and samples with various dilutions were analyzed using the Friedman test and post-hoc Wilcoxon comparisons with Bonferroni correction. Data for hematocrit and platelet counts are reported as mean ± SD. Data for ROTEM and PFA are displayed as median (interquartile range). Significance was set at *P* < 0.05.

## Results

### Hematocrit and platelet counts

The complete blood counts revealed a mean hematocrit of 49 ± 3.4 % (0.49 ± 0.034 l/l) and mean platelet count of 252 ± 41 × 10^9^/l.

### Platelet Function - PFA

Baseline values of the 15 dogs were within the reference range reported by Callan and Giger (52–86 s) [31], except for one dog, which had a baseline value of 88 s.

**Fig. 1:**
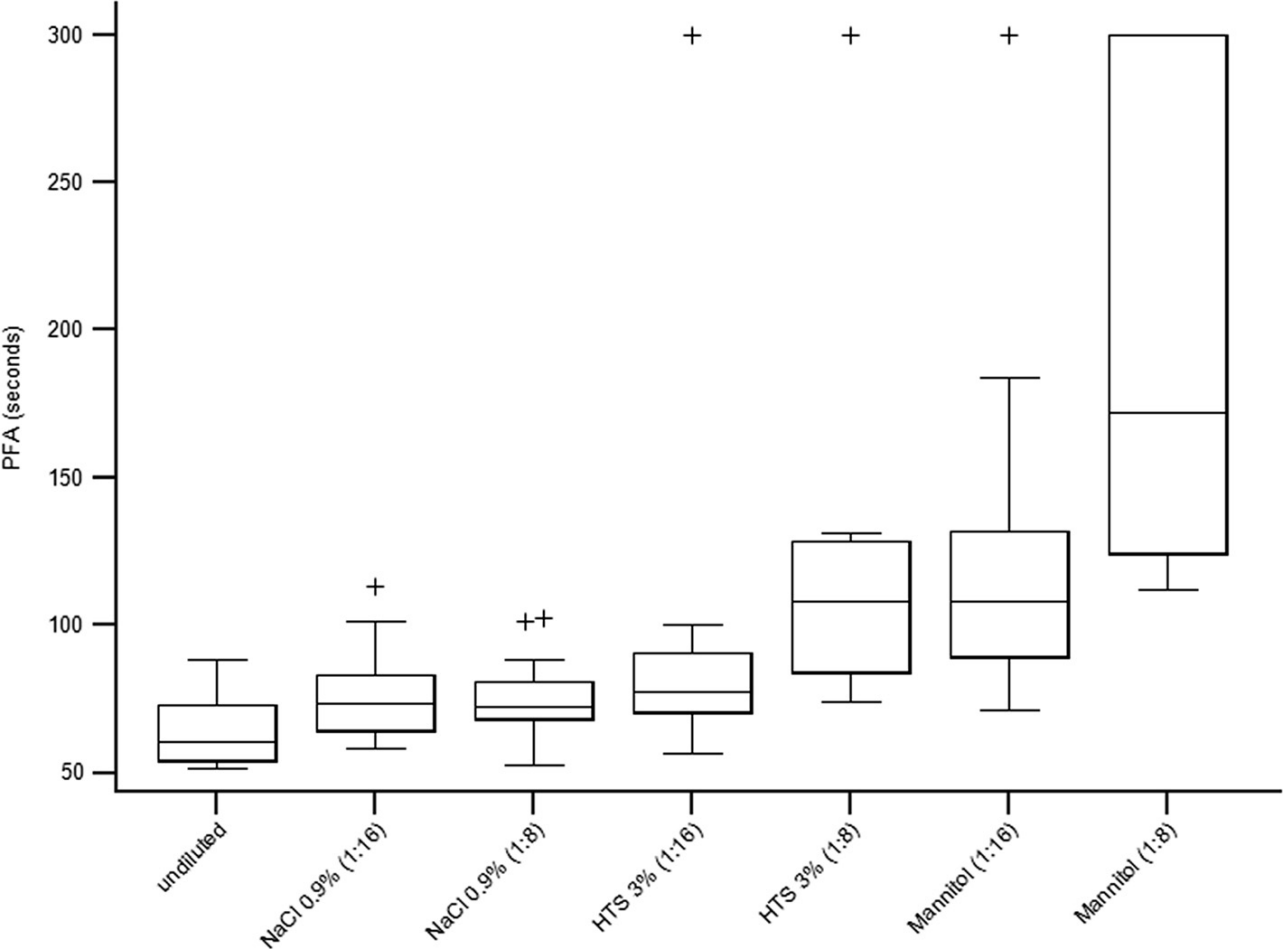
Box plots showing platelet function analyzer closure times (Ct_PFA_) in undiluted blood samples and samples diluted with 0.9 % saline, 3 % HTS, and 20 % mannitol in 1:16 and 1:8 dilutions

A significant difference was found between samples for Ct_PFA_ (*F* = 28.5, df = 6, *P* < 0.001) (Table 1, Fig. 1). Pairwise comparisons revealed that, for 3 % HTS, only the high dilution (1:8) resulted in significant prolongation of Ct_PFA_ compared to baseline (*P* = 0.002) or saline (*P* = 0.002). In contrast, both dilutions with 20 % mannitol resulted in significant prolongation of Ct_PFA_ compared to baseline (*P* = 0.002). In addition, the high dilution resulted in significant prolongation of Ct_PFA_ compared to both saline (*P* = 0.002) and 3 % HTS (*P* = 0.008) (Table 1, Fig. 1).

**Table 1:** Thromboelastometry and platelet function analyses in undiluted blood samples and samples diluted with 0.9 % saline, 3 % hypertonic saline, and 20 % mannitol in a ratio of 1:16 and 1:8 in 15 dogs

Variable	Baseline (undiluted)	0.9 % NaCl (dilution 1:16)	0.9 % NaCl (dilution 1:8)	3 % HTS (dilution 1:16)	3 % HTS (dilution 1:8)	20 % mannitol (dilution 1:16)	20 % mannitol (dilution 1:8)
PFA-100							
Ct_PFA_ (s)	60 (51–88)	73 (64–83)	72 (68–81)	77 (70–90)	108 (84–126)*^,^**	108 (89–132)*	172 (124–300)*^,^**^,^***
Ex-tem							
CT (s)	35 (28–55)	38 (36–60)	44 (31–62)	37 (33–83)	40 (34–48)	51 (38–99)*	90 (46–134)*^,^***
CFT (s)	149 (131–159)	151 (128–184)	151 (123–175)	186 (148–206)	196 (176–249)*^,^**	204 (158–249)*	252 (222–314)*^,^**^,^***
MCF (mm)	59 (55–62)	56 (50–60)	55 (53–57)	54 (51–57)	50 (46–53)*^,^**	53 (51–56)	49 (45–50)*^,^**
Fib-tem							
CT (s)	38 (30–43)	41 (33–51)	38 (32–47)	36 (32–42)	36 (32–43)	54 (28–66)	48 (38–58)
MCF (mm)	8 (8–10)	7 (5–9)	7 (6–8)	7 (6–9)	7 (6–9)	7 (4–9)	5 (4–5)*^,^**^,^***

Values are presented as median (interquartile range) *significant difference (*P* < 0.05) compared with baseline; ^**^significant difference (*P* < 0.05) compared with 0.9 % saline at the same dilution; ^***^significant difference (*P* > 0.05) compared with 3 % HTS at the same dilution. *Ct*_*PFA*_ closure time, *CT* clotting time, *CFT* clot formation time, *HTS* hypertonic saline, *MCF* maximum clot firmness

### Whole blood coagulation - ROTEM

Baseline values of the 15 dogs were within reference ranges reported by Falco at al. [32] and Smith et al. [29]

#### Ex-tem CT

**Fig. 2:**
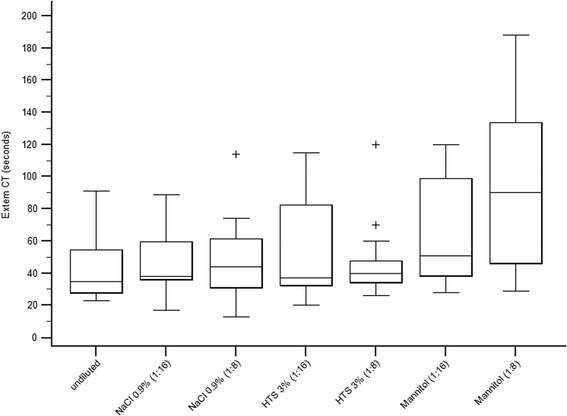
Box plots showing Ex-tem CT measurements in undiluted blood samples and samples diluted with 0.9 % saline, 3 % HTS, and 20 % mannitol in 1:16 and 1:8 dilutions

A significant difference was found between samples for Ex-tem CT (*F* = 5.8, df = 6, *P* < 0.001) (Table 1, Fig. 2). Pairwise comparisons revealed that only samples diluted with 20 % mannitol in both the 1:16 dilution (*P* = 0.006) and 1:8 dilution (*P* = 0.019) differed significantly from baseline. In addition, Ex-tem CT was increased in samples with 20 % mannitol compared to 3 % HTS in a 1:8 dilution (*P* = 0.042) (Table 1, Fig. 2).

#### Ex-tem CFT

**Fig. 3:**
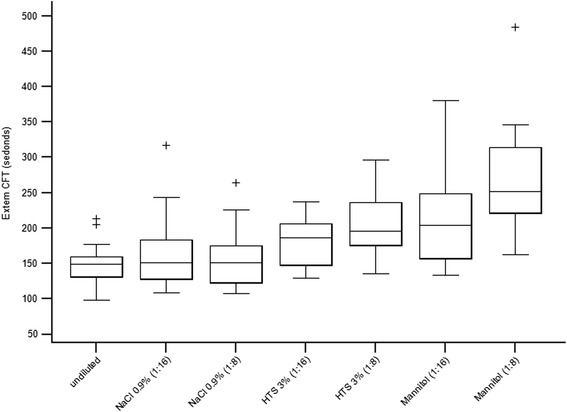
Box plots showing Ex-tem CFT measurements in undiluted blood samples and samples diluted with 0.9 % saline, 3 % HTS, and 20 % mannitol in 1:16 and 1:8 dilutions

A significant difference was found between samples for Ex-tem CFT (*F* = 28.6, df = 6, *P* < 0.001) (Table 1, Fig. 3). Pairwise comparisons revealed significant differences from baseline in samples diluted with 3 % HTS, 1:8 dilution (*P* = 0.004), and with 20 % mannitol, 1:16 dilution (*P* = 0.004) and 1:8 dilution (*P* = 0.002). Compared to saline, Ex-tem CFT was only significantly more prolonged in samples with 1:8 dilutions of both 3 % HTS (*P* = 0.002) and 20 % mannitol (*P* = 0.002). In addition, Ex-tem CFT was more prolonged in samples with 20 % mannitol compared to 3 % HTS in 1:8 dilutions (*P* = 0.002) but no significant difference was found between the samples in 1:16 dilutions (Table 1, Fig. 3).

#### Ex-tem MCF

**Fig. 4:**
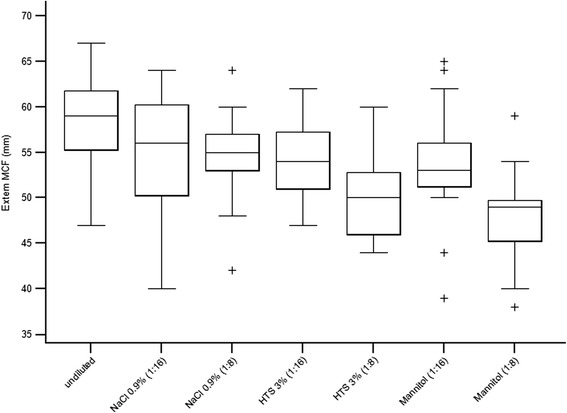
Box plots showing Ex-tem MCF measurements in undiluted blood samples and samples diluted with 0.9 % saline, 3 % HTS, and 20 % mannitol in 1:16 and 1:8 dilutions

A significant difference was found between samples for Ex-tem MCF (*F* = 22.6, df = 6, *P* < 0.001) (Table 1, Fig. 4). Pairwise comparisons revealed that Ex-tem MCF was reduced in samples with 1:8 dilutions of 3 % HTS (*P* = 0.002) and 20 % mannitol (*P* = 0.002) compared to baseline. In addition, a reduced MCF was found in 1:8 dilutions of samples with 3 % HTS (*P* = 0.004) and 20 % mannitol (*P* = 0.002) compared to saline. No difference was found between 3 % HTS and 20 % mannitol in either dilution, or between saline, 3 % HTS or 20 % mannitol in 1:16 dilutions (Table 1, Fig. 4).

#### Fib-tem CT

No significant differences were found between samples for Fib-tem CT (*F* = 1.90, df = 6, *P* = 0.093) (Table 1).

#### Fib-tem MCF

**Fig. 5:**
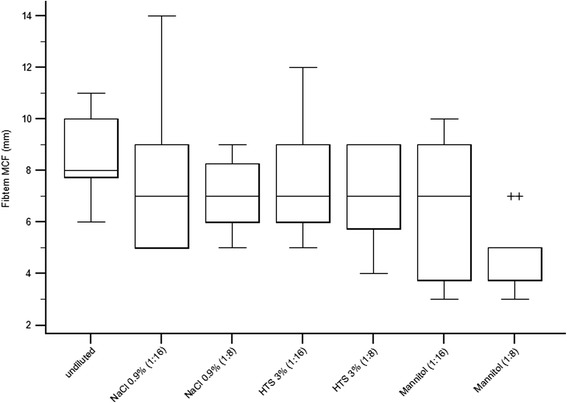
Box plots showing Fib-tem MCF measurements in undiluted blood samples and samples diluted with 0.9 % saline, 3 % HTS, and 20 % mannitol in 1:16 and 1:8 dilutions

A significant difference was found between samples for Fib-tem MCF (*F* = 12.3, df = 6, *P* < 0.001) (Table 1, Fig. 5). Pairwise comparisons revealed a significantly reduced MCF in samples with 1:8 dilution of 20 % mannitol (*P* = 0.004) compared to baseline. In addition, Fib-tem MCF was significantly reduced with 1:8 dilution in samples with 20 % mannitol compared to 3 % HTS (*P* = 0.004) and saline (*P* = 0.021) (Table 1, Fig. 5).

## Discussion

Thromboelastometry using ROTEM is a dynamic analysis of specific aspects of the coagulation cascade including clot formation, clot strength, and subsequent fibrinolysis [29]. As platelet function is inhibited by cytochalasin-D in the Fib-tem assay, this assay permits assessment of fibrin polymerization alone. These methods therefore provide information about the platelet function, the interaction of platelets with the coagulation cascade, fibrin polymerization, and clot lysis.

Both mannitol and HTS solutions are recommended for the treatment of intracranial hypertension in dogs [2]. Results of the present study revealed that in vitro dilutions of canine blood with 3 % HTS and 20 % mannitol, but not with saline, affected ROTEM and PFA parameters in a dose dependent fashion. Some of these effects were only evident between samples diluted with 3 % HTS or 20 % mannitol and undiluted samples. However, effects on the measured parameters were also noted when comparing samples at the high dilution (1:8) with 3 % HTS or 20 % mannitol and those diluted with saline. These were an increased Ct_PFA_, prolonged CT and decreased MCF in the Ex-tem profile in samples diluted with 3 % HTS, and prolonged Ct_PFA_, CFT Ex-tem, and decreased MCF in the Ex-tem and Fib-tem profiles in samples diluted with 20 % mannitol. In addition, dilution with 20 % mannitol lead to a prolonged Ct_PFA_, CT, and CFT in Ex-tem, and a decreased MCF in Fib-tem compared to 3 % HTS at the high dilution.

The dilutions selected were chosen to mimic clinical doses, assuming canine blood volumes of approximately 80 ml/kg [33]. The recommended initial bolus dose for the treatment of intracranial hypertension is 5 ml/kg for both 3 % HTS and 20 % mannitol (1 g/kg mannitol), which corresponds to a 1:16 dilution [2], and a 1:8 dilution was chosen to examine changes at twice the initial recommended bolus dose.

As both solutions are of similar osmolarity (20 % mannitol, 1098 mOsm/l; 3 % HTS, 1072 mOsm/l), differences between solutions were unlikely due to osmolarity alone. The possible effects of HTS and mannitol on a cellular level were not evaluated in the current study, and flow cytometry or electron microscopy of platelets might have added important information in regard to platelet activation and platelet structure. Although 20 % mannitol impaired blood coagulation to a greater extent than 3 % HTS, this was only apparent in the high dilution (1:8), and no clear advantage of either solution was found with regards to coagulation effects at the dose selected to mimic a single bolus (1:16 dilution). Indeed, in the present study many values were significantly different from baseline and/or the dilutional control, but an effect beyond the reference ranges reported by Smith at al. [29] were only found after the high dilution (1:8) with 20 % mannitol for two parameters (CT and CFT Ex-tem) and with 3 % HTS for one parameter (CFT Ex-tem). Data in the present report does not therefore support any clear advantage of one osmotherapeutic over the other. Nevertheless, findings in this study appear to corroborate results from two previous in vitro human studies, which demonstrated hypocoagulability caused by 15 % mannitol that was more pronounced than that caused by 2.5 % HTS or by a combination of hydroxyethyl starch with 15 % mannitol using ROTEM [12, 34].

A limitation to the present study is that all blood samples were collected from healthy dogs and the analyses were performed in vitro. The effects of osmotherapeutics on coagulation may be more pronounced in dogs with pre-existing coagulopathies, and may also significantly differ in vivo. Further studies are therefore necessary to evaluate the true in vivo risk of hemostasis impairment following administration of these agents in dogs with intracranial hypertension and/or pre-existing coagulopathies.

Investigations into the pathophysiology of coagulation impairing effects of hyperosmolar solutions are rare. Possible mechanisms for platelet dysfunction after hyperosmolar therapy include osmotic shock and reduced platelet calcium mobilization required for triggering platelet activation [35, 36]. In a recent human in vitro study, hypertonic hydroxyethyl starch caused platelet dysfunction, and deformed, activated and severely aggregated platelets, suspected due more to the HTS component than the colloid component of the solution [15]. In addition, hypernatremia may impact plasmatic coagulation [14, 37], and impaired enzymatic function in the clotting cascade resulting from an increased sodium ionic strength may contribute to hypocoagulability [13]. Similar to other hypertonic solutions, mannitol may cause hyperosmotic stress, resulting in changes in platelet function and coagulation [14]. Lastly, recent research suggests that both 7.5 % HTS and mannitol inhibit agonist-induced CD40L expression on platelets, which is a marker of platelet activation and inflammation [38].

Besides specific effects on coagulation, a certain degree of dilutional coagulopathy may be expected after hypertonic fluid administration. In vivo, this effect is due to transcellular fluid shifts from the extravascular to the intravascular space due to the osmotic pull of intravascular hypertonic agents (approximately 7 milliliter of free water for every milliliter 7.5 % hypertonic saline infused) [37]. These shifts result in increased intravascular volume and subsequent dilution of the administered agent and reduced hypertonicity, as well as dilution of clotting factors and platelets. Given the in vitro nature of the present study, no redistribution of fluid can occur, resulting in dilution of clotting factors and platelets without reduction of hypertonicity. Moreover, in vivo processes, such as buffering and electrolyte homeostasis cannot occur. The results of this and other in vitro studies can therefore only approximate in vivo conditions and the potential clinical impact on hemostasis.

A further limitation to this study is that in vitro blood dilutions of 10 and 20 % with mannitol have previously been found to significantly decrease both hematocrit and platelet counts in humans [39]. As Ct_PFA_ may be prolonged by a hematocrit below 25 % or platelet counts below 100,000 platelets/μL, the observed effects of mannitol on Ct_PFA_ may be due only to hematocrit or platelet lowering effects [31]. In contrast, blood dilution resulting in decreased hematocrit is expected to result in hypercoagulable ROTEM tracings [39]. As platelet counts and hematocrits following dilutions were not measured in the present study, the degree to which these may have affected results is unclear. Moreover, the dilution of citrated blood samples with the citrate-free study solutions may have resulted in increased calcium concentrations following recalcification in the ROTEM cup. This in turn could impact thrombin generation. However, as only minor changes were observed following dilution with saline, these effects were unlikely to impact results of this study.

Additional limitations to the present study relate to sample handling and stability. One limitation was that some of the ROTEM measurements were started 2.5 h after blood sampling due to the numbers of samples evaluated from each blood draw. This is considerably longer than the 30 min recommended by Smith et al. [29]. Indeed, prolonged storage led to hypercoagulable TEG results in dogs in a previous study [40], because canine blood undergoes significant ex-vivo contact activation during and after blood collection [29]. This prolonged storage time may have affected ROTEM results in the present study. A further limitation was that samples were stored at room temperature (24 °C) and may have taken several minutes to rewarm to 37 °C when added to the pre-warmed thromboelastometry cup [29]. This could result in temperature inconsistencies, affecting ROTEM results. Lastly, duplicate measurements could have mitigated some of the effects of variabilities in sample handling on ROTEM results, but this was not performed in the present study as it would have doubled sample storage time.

## Conclusion

In vitro dilution of blood with both mannitol and HTS caused hypocoagulability and platelet dysfunction in a dose-dependent fashion. The data in this study suggest that at high dilutions mannitol causes a slightly greater impairment of whole blood coagulation and platelet function than HTS. However, at low doses, simulating a clinically recommended dose, no difference was found in coagulation effects between the two solutions. The results of this in vitro study are difficult to extrapolate to an in vivo situation because important elements such as transcellular and vascular fluid shifts, and the intravascular and interstitial spaces cannot be assed with an in vitro model. Further studies on blood samples from dogs following intravenous administration of these solutions are necessary before recommendations can be made.

## Abbreviations

CFT: Clot formation time
CT: Clotting time
Ct_PFA_: Platelet closure time
HTS: Hypertonic saline
MCF: Maximum clot firmness
PFA-100: Platelet function analyzer-100
ROTEM: Rotational thromboelastometry
